# A Treatise on Therapeutical Hygiene

**Published:** 1861-11

**Authors:** 


NORTH AMERICAN

MEDICO-CHIRURGICAL REVIEW.

NOVEMBER, 1861.

### Analytical and Critical Reviews.

ART. I.—*Traité d'Hygiène Therapeutique, ou Application des Moyens de l'Hygiène au Traitement des Maladies.* Par F. RIBES, Professeur d'Hygiène à la Faculté de Médecine de Montpelier, etc., etc. Paris: J. B. Baillière et Fils. 8vo., pp. 828.

*A Treatise on Therapeutical Hygiene, or the Application of the Means furnished by Hygiene to the Treatment of Diseases.* By F. RIBES, etc., etc.

HYGIENE was the companion, if not the precursor, of ancient Greek medicine, and its importance in this connection was admitted by the most distinguished Roman physicians. During the long period of intellectual darkness which followed the downfall of Rome and that of the scholastic subtleties and jargon which succeeded this latter in its turn, hygiene was neglected alike in the usages of ordinary life and in theoretical teaching. When, eventually, it found a place in the didactic courses of the medical schools of the last century, it remained, in a great degree, isolated and barren, or, at most, was made up of a series of commonplaces, which were inefficient either for the preservation of health or the cure of disease. By a large majority of English and American physicians hygiene had come to be regarded as a subject of pure lore, which one might occasionally discuss in a spirit of curiosity, but ignorance of which was not discreditable, on the score either of learning or of practical knowledge. It is within the present half century since the writer of these remarks acquired among the students of medicine to whom he had begun to lecture, the soubriquet of "hygiene," so little, if at all, had they heard of the subject, or of its being available, in their future professional career, for the successful treatment of their patients.

The daily impediments to the cure of diseases, owing to the ignorance and consequent neglect of the principles of hygiene, ought, one might suppose, to have prevented the attention of the commonest observer from being ever dormant on agencies constantly operative. But men continued, notwithstanding, to be somnolent, or to look with stupid indifference on all the physical evils with which they were surrounded, so far as health alone was concerned. When ameliorations and improvements were made, it was to gratify the senses in the vanity of show and of luxury. Habit, indolence, a secret belief in fatalism, while, perhaps, openly disavowing it, cupidity, which grudges a present outlay when health, not market profit, is the only promised return—all these causes kept hygiene in abeyance.

Commerce and manufactures, by accumulating large populations in limited and confined spaces, and placing men in new and often unhealthy relations with things around them, at last awakened a certain number of outside observers, more indeed than those actually engaged in these pursuits, to a perception of the heavy tax in disease and shortened duration of life paid for carrying them on. Modern wars also, by bringing together large masses of men under new and peculiar circumstances of location, atmospherical exposures, and bad or defective nutriment, and close lodgings, furnished opportunities for the study of hygiene, which could not be overlooked, although they were seldom turned to full account by medical men connected with the army and navy. Of late years, however, precious documents have been prepared by these parties, full of instruction, albeit of a melancholy kind, since they reveal the inaptness and obstinate adherence to old prejudices and semi-barbarous usages of those in power, who have had control of armies and fleets.

If the interest felt in a subject bears any definite proportion to its importance, then ought hygiene, which includes the questions of life, health, and longevity, to command unceasing attention. Looking at the comparative influence, constant operation, and abiding effects of hygienic agents on the one hand, and the restricted and occasional employment of those purely medicinal or pharmacological on the other, the first might be supposed to be the subject of hourly meditation and study by every intelligent human being; while the second, as only of occasional use and adaptation, would enlist for their investigation the minds of a limited number of persons. But, in fact, the proportions in the two cases are reversed, for while there is a limited class, called doctors, who make the operation of drugs and the cure of disease their special study, yet everybody has a medical dogma, talks more or less of disease, and of some favorite remedy for its cure. On the other hand, health and the means of preserving of it are treated, when thought of at all, as a secondary matter, the inquiries into which are made by a small number, whose teachings are coldly listened to. This must seem the more strange when we reflect that health, to a certain extent an original gift, can only be preserved, or, if temporarily lost, can only be regained by obedience to the laws of hygiene. Is the digestion to be improved, new and better blood to be elaborated, fresh tone to be imparted to the nervous system, and the muscular tone to be developed and strengthened, we seek for the means of obtaining these desirable results by bringing the functions into harmonious relations with the several hygienic exciters, by which they are habitually stimulated and nourished, viz., food, air, exercise, bathing, due division of the hours of waking and sleep, grateful appeals to the senses, and proper exercise of the mental faculties. Would we alter the diathesis or constitutional predispositions to certain diseases, such as the scrofulous and the scorbutic, it would be worse than time lost to seek for the means of doing so in the use of drugs, or of medicines properly so called. Equally futile would be the attempt, when the diathesis has been converted into actual disease, to cure scrofula and scurvy by remedies derived from the shop. The causes of the disease, in either case, consist in a withholding of certain necessary hygienic agents —pure and dry air, wholesome food, made up in due proportion of animal and vegetable substances, and pure water for drink: the cure will be brought about by a regular and persistent use of these several agents. Who but the veriest dreamer looks for the prevention and cure of pulmonary consumption by medicine? Who does not rest his hopes solely on judicious hygienic treatment, which includes change of climate, travel, active locomotion, food, and appliances to the skin, by proper clothing, frictions, and bathing? If we put aside the sophistries suggested by perverted appetite and long habit, we must see that there is no correction of the gouty and rheumatic diathesis, or radical cure of gout and rheumatism by medical treatment alone, whether attempted by the use of so-called specifics, or carried on after the most approved practice of the schools. Our reliance must be on the methodical and persistent use of a suitable regimen in its most comprehensive sense. Even in febrile diseases, as those of the eruptive class, which are produced by the introduction into the organism of a specific poison, we are beginning to realize the fact that less is to be expected from medicines than from hygienic means—fresh air of a regulated temperature, large dilution, by cool drinks, and ablution or bathing in water of a temperature dependent on that of the skin; the more elevated the latter the cooler the former. We have no better means than these for eliminating the poison by the various secretions, and of moderating the violence of its action on the organs. The promises of chemistry, as a therapeutical guide, so far have not been realized.

In our preceding remarks we have looked at the subject in a synthetical point of view, and at the results of hygienic agents in the aggregate. An analytical inquiry into the operations and effects of each separate agent on the organism, will be found in the work the title of which heads this paper. The investigations of M. Ribes might have been more searching and connected with a larger amount of historical experience than he has thought proper to introduce; but, viewed as a whole, this treatise on therapeutical hygiene is a valuable contribution to a study which, notwithstanding its antiquity, has scarcely acquired a regular shape or well-defined outlines. The author begins by speaking of the nature of therapeutics—the indications, methods, means, and direction. "The means which tend to cure are, first, actions of the animal economy which are endowed with a conservative and medicating power; secondly, hygienic agents; thirdly, pharmacological agents; fourthly, surgical agents." That part of the practice of medicine which is here called *therapeutical hygiene* was designated by the old writers under the name of *dietetics*, but as this latter term is restricted now to that which relates to alimentary regimen, the former, as more comprehensive in its meaning, would seem to be entitled to the preference.

Under the head of "therapeutical means," we are told that

"From the time of Hippocrates, even from that of the Pythagoreans down to Galen, they constituted the chief part of the resources of practical medicine. The Greek school placed their incomparable value in the clearest light. Hippocrates especially attached more importance to dietetics than to medicines, of which latter he made a very measured use. Many physicians before his time only employed these latter externally. At a subsequent day Celsus and Galen laid down rules of regimen which might suffice even now for the guidance of physicians in the larger number of cases."

Hygienic agents, by their abrupt or excessively prolonged action, are frequently predisposing causes of disease, and yet, if wisely modified in their application, they will produce a change both positive and permanent in one or more functions, and bring back the entire economy to its physiological or normal rhythm. The reaction against ancient medicine, brought about by Paracelsus and his followers, the readiness to believe in the promises of empirics, the progress of natural history and of materia medica, opened a wide field for the trial of new substances, and caused nature to yield her supremacy to art. A preference was given for the means which were capable of acting powerfully and promptly. It took a long time to discover, and still longer to make the acknowledgment, that confident promises were often illusory, and that great vogue ended in discredit. Undoubted success in some instances, and valuable discoveries, led to a blind trust in active medication. But all this did not justify the putting aside olden experience, and the simple, wise, and prudent practice which confided in the powers of the animal economy and of therapeutical hygiene.

It is well for us to bear in mind, however, that, at all times, great practitioners of medicine paid special attention to therapeutical hygiene, and knew how to turn it to rational account. Ambrose Paré, the father of French surgery, used to say that it was better to come out of disease by the aid of a good regimen than by medicines, which are disagreeable to take, hard to retain, and painful in their operation. Diet and quiet have often performed wonders, both in warding off the imminent attack of virulent disease, and, in other cases, in curing it after it has come on.

The means of acting on a morbid state of the organism and bringing about in it the desired changes, are—1. Through the functions of nutritive life, and especially the digestive, the respiratory, the excretory, and the secretory functions. 2. By means derived from the affective or moral functions. 3. By those from the intellectual life. 4. By those drawn from the physical life, or that of action.

The first division opens with the subject of "Alimentation in general." The effects obtained by the aid of alimentary regimen depend, on the one hand, on the quantity of the nutritive substance, its nature, and that of the condiments with which it may be seasoned and the processes to which it is subjected; on the other hand, to the temperament, constitution, habits, and age of the patient. The effects of the food taken will, also, be modified by the nature of the disease. An inquiry into all these points becomes the more important from the fact that death is often caused by errors in regimen. Among the greatest obstacles in carrying out a dietetic system, are the capricious appetites and tastes of the invalid, and the mischievous, although well meant, recommendation of his friends, concessions to which are too readily made by the physician.

Abstinence, or "a very strict diet," is studied in the next chapter, in reference to its use in acute diseases. A physician of the last century said that he left behind him two excellent doctors—diet and water. Another used to dwell on the skill of Doctors Diet and Quiet. Going back to the fountain head, we might cite the dictum of Hippocrates, that he was the most skillful physician who cured by means of regimen. This acute observer established many degrees in *diet:* from the entire privation of food, or "extreme diet," to the use of food consisting of solid vegetable and animal substances. In acute diseases the recuperative powers of the economy are sufficiently strong to do without aid of an exciting nature, which would increase instead of relieving the existing oppression of the patient. Hunger, says the sage whom we have just quoted, exerts great power: it weakens, it cures, it kills. Without it a great many acute diseases would be necessarily fatal. An abatement of the customary activity of the digestive system is followed by that of the other parts of the organism, which sympathize with and derive their support from it. Both the general and the local irritation are diminished by this means, and the organism supports better the shock of the disease. Fluxions are removed and congestions diminished, for abstinence or a very reduced diet increases interstitial absorption at the same time that it aids the various excretions.

Celsus tells us, that Asclepiades forbade all kinds of nutriment to his patients during the first three days of acute diseases; and, following his example, Themison adopted this practice in the greater number of the diseases which he was called upon to treat. But, as justly remarked by M. Ribes, no rule can be laid down with mathematical precision in these cases; we must look to the different nature and degrees of intensity of constitutional actions. We shall not follow closely the author in his dietetic directions, which are blended with, if not sometimes obscured by, doctrinal views of the older writers. We could wish, also, that he had spoken, more than he has done, from personal observation, and given us the hygienic results in modern clinical practice. He describes strict diet to be that which consists exclusively of drinks and of aliments in a fluid state, easy of absorption, and tasking but moderately the organs of assimilation. It is not only the amount of the matter or of the molecules contained in these substances, but also their nature, which must direct their selection by the physician. The rules for the regulation of this strict diet in acute diseases are subordinate to their *periods* and to their *nature*. The most favorable time in disease for taking food is during the intermission. Another important element in solving this problem is judiciously adverted to by the author. It is the influence of the nervous system or of the feelings and emotions of the patient. The food may be selected with the nicest discrimination and prepared in the most approved manner, and yet, if the patient be exposed to mental irritation or annoyance of any kind, he will suffer from indigestion and increase of his morbid state. The surroundings of the sick person are, therefore, every way important, so as to keep away intruding and unasked-for visitors, and avoid the introduction of disagreeable topics. There is yet another very urgent reason for watchfulness in guarding the patient from harm, committed under the guise, and really with the good intentions, of friendship. Many kind but ignorant souls, both in the house and out of it, who, confounding apparent weakness caused by the first violence of the disease with that from exhaustion growing out of its long duration, imagine that nothing better can be done than to keep up the strength, as they call it, by nourishing food and diffusible stimulants—wine, brandy, etc. We would add, that the patient often labors under similar delusion, and is too ready to take from the offering hand these articles, which are all calculated to aggravate the fever or the inflammation and increase the debility. Were the instinctive cravings of a febrile patient, instead of a false theory, to be the guide, no food would at first be asked for, and cool, acidulous drinks would be alone required. Animals have often a better chance of recovery than human beings, as the former yield to their instincts and do not force themselves to take food, but wait until the natural appetite returns with the abatement or removal of the disease.

In a subsequent article, on the application of the rules of regimen to the treatment of a certain number of internal diseases, we meet with the following paragraph, describing the therapeutical treatment of pneumonia by an eminent teacher and reformer. It will, we are afraid, shock the anti-bleeding and liquor-prescribing creed of certain writers and practitioners of the present day.

"Among our contemporaries, Broussais is one of those who have turned dietetics to the best account in the phlegmasiæ, both acute and chronic. 'In vain,' said he, 'will you enlist the most appropriate means for removing *inflammation of the lung;* if the regimen does not concur in this end, they will be almost always without effect. While we are protecting the skin against the impression of cold, while we are withholding all mechanical or chemical irritants, and preventing all sensations and acts which might produce excitement, while we are bleeding and giving sedative drinks, and mildly fomenting the skin, or irritating it either to produce rubefaction or phlogosis, or to create a suppurating sore, we must regulate attentively the regimen. The most strict diet ought to be prescribed during the first period of a violent inflammation; but when it becomes chronic, there will be less occasion for restriction. Affections of the lungs may be treated almost exclusively by diet. If the phlogosis is simple, it will suffice to subject the patient to a diet (abstinence) as rigorous as that pursued in the anti-aneurismal treatment of Valsalva. If, at the same time, the other antiphlogistic means are applied with judgment, we shall find in a short time the lung restored to all its functions.'"

We ought to have repeated, after M. Ribes, the directions which he lays down in a preceding page for the administration of demulcent, diluent, and cooling drinks, as constituting the regimen in the first stage of acute disease.

"Until the date of the resolution of the phlegmasia, the indication is to diminish the exaltation of the morbid state by aqueous, gummy, or mucilaginous drinks, slightly sweetened. These drinks should be given tepid, or even cool, in small quantity at a time, as the irritated stomach is inclined to vomiting. After the demulcent substances come vegetable acids diluted with water and sugar, viz., orangeade, lemonade, currant, or raspberry water. The agreeable sensation of coolness which they procure, and the direct antiphlogistic or temperate operation which they induce, will be followed by excellent effects, if the patient make moderate use of them.

"This strict diet will be continued so long as the local symptoms and febrile movement are intense and persistent. When they have ceased, recourse will be had to barley, rice, apple or prune water, chicken water or a decoction of veal—substances which are somewhat more nutritive than the preceding. The taste of the patient will be consulted in the choice of these articles."

Melancholy proofs of the necessity of abstinence in acute diseases are too often furnished by the deaths which have followed a neglect or a breach of the rules enjoined in this particular. Instructive remarks are made on the most appropriate regimen at the termination of acute diseases, and in greater detail during convalescence. The completeness of this latter will depend greatly on the choice of aliments. The quantity as well as the nutritious quality of food is to be gradually increased in convalescence, according not only to the appetite, but to the ability and readiness of the stomach to digest it.

In the chapter on dietetics in chronic diseases, we meet with much discriminating advice. "Hippocrates, Celsus, Aretæus, Galen, among the ancients; Sydenham, Baglivi, Tissot, Broussais, among the moderns, have furnished excellent data for the solution of the problem of the application of regimen to chronic diseases." Most practitioners tell us that the functions of the digestive canal are deranged in these cases. Zimmerman advises that we should direct our attention to the state of the stomach and bowels.

"We must," he says, "restore the digestion if we would succeed in curing lingering diseases. The medicines indicated and properly prescribed will doubtless contribute to the end proposed; but the precautions exacted by the constitution or state of the vital powers, the nature of the disease, and its duration point more to the resources furnished by dietetics than by pharmacy. It is only by a union of the two that the results will be satisfactory. Nothing will be so dangerous as aliments given unseasonably or without precise rules. We frequently see in the course of chronic diseases accidents or complications; vomitings, diarrhœa, dysentery even, dropsies, emaciation, which have no other cause except badly directed alimentation."

Looking for the causes of chronic diseases in connection with errors in regimen, we find them designated as follows: "Excess in the use of alcoholic drinks is one of the exciting causes of insanity and other cerebral affections, chronic inflammation of the intestinal canal and liver, and dropsy which follows in their train. Insufficient alimentation or that of a bad quality gives origin to chronic diarrhœa and various asthenic maladies, to scurvy, and intractable intermittent fevers. The exclusive use of farinaceous aliments engenders scrofula and keeps up asthenic diarrhœa.

"An altered alimentary substance produces pellagra, and penury favors its effects. It is not the Indian-corn itself, but the grain altered in its character, which causes this disease. This change is owing to the presence of a fungoid parasite, which is frequently met with in northern Italy.

"*Ergot,* an excrescence which is developed on the end of the grain of rye, gives rise to diseases of extreme gravity.

"Hygienic treatment and the chief means of preventing, diminishing, and curing the disease consist in a change in the food."

The great number of disorders seen in prisons is due to the influence of badly directed alimentation, and of the punishments inflicted on the prisoners, which go to diminish the allowance of food already too restricted in quantity.

"The diseases most commonly met with in prisons are the various forms of scrofula and phthisis pulmonalis. It is to be observed at the same time that the antecedents of these diseases consisted in a life of destitution and vice. A third disease is scurvy—the appearance of which is readily explained by the prison food, vitiated air, and want of cleanliness in clothing."

Excessive bodily labor, disproportionate to the strength, is followed by general asthenic diseases, especially noticeable in children who work in factories. Suitable alimentary regimen will fulfill one of the chief indications for the cure of these diseases.

"Finally, the abuse of mental labor, and the bad hygienic course of those who give themselves up to it, is a cause of disorder of the stomach and other abdominal viscera, as well as of the entire economy, which regimen may abate if it does not entirely remove the effects."

Age, hereditariness, and temperament are modifying conditions in carrying out a suitable regimen, which must never be lost sight of. More than all, are the considerations depending upon the *periods of the disease.* On this point we do not find very clear or precise directions laid down by M. Ribes, who every now and then is lost in the abstract vitalism and corresponding terminology with which the school of Montpelier has always been affected.

Alimentary agents, considered in relation to the treatment of chronic diseases, is the subject of the fourth chapter of the first book in the volume before us. Abstinence is first considered. A remark made by Baglivi deserves to be repeated. It is, that persons who were cured of their chronic maladies during Lent, under the influence of fasting and vegetable aliments, suffered from relapses at Easter, when the use of meats was allowed. Chronic engorgements, induration, hypertrophy, organic alterations or degenerations are decidedly benefited by abstinence; so, also, are ulcers with superabundant serous suppuration. Gout has disappeared, and sometimes been entirely removed by the persons subject to this disease having been made prisoners of war and kept on bread and water. But, adds the author, abstinence alone does not ordinarily cure the gout.

Under the title of *cura famis,* or cure by hunger, Galen, and at a later period Valsalva and Callisen, designate a mode of treating certain diseases in which entire abstinence—still proportioned, however, to the state of the patient, who is not to be thrown into utter prostration—constitutes the chief part of the treatment. Récamier made use of it, added to the large use of cicuta, in cancer. He trusted exclusively to these means, whereas Valsalva had recourse, in addition to the *cura famis* for the treatment of aneurism, to free blood-letting. The quantity of food and drink was daily diminished, so that at last only eight ounces of panada made up the morning meal, and half this quantity was given in the evening. So soon as the patient was sufficiently attenuated to the point of his being scarcely able to raise his hand from his bed, to which he had been confined from the beginning, the quantity of food was increased each day in exceedingly minute proportions until he was able to rise. In our own time, Marjolin and Bérard assert, that when false consecutive aneurisms cannot be compressed, and when their situation is such as to render ligation dangerous, recourse may be had to the simultaneous employment of topical refrigerants and the method of Valsalva.

The *Arabic* regimen is described by the author. It consists in a dry diet, which few patients will deserve their name by persisting in. Cases in proof of its efficiency in different diseases are cited by Aristotle and Pliny, and among the moderns by Schenck and Buchan. The latter says that the sick person must abstain from all kinds of drink, especially the aqueous, and be content to restrict himself to dried bread, sea biscuit, meats and jellies, and aromatic and stimulating vegetables. The thirst may be mitigated by a few mouthfuls of whey or Seltzer water, or of wine rendered diuretic. Of late years Dr. C. J. B. Williams recommends the dry regimen in catarrh. He cites his own case in its favor. On an average, forty-eight hours of abstinence from liquids will be quite enough to effect a cure. The period may be shortened by exercise and warm clothing, or by lying warm in bed, or by commencing with a purgative, or by any other dry means of increasing the natural secretions.

Analogous to this kind of regimen is that which consists in the exclusive use of sugar, for this substance is not a constitutive one, or if at all, it is so in but a slight degree, by a little adherent mucilage, which it is the object of refinery to remove. It has no recognized therapeutical action. Garnier, of Guadaloupe, is quoted by Lullier Winslow as having cured many cases of dropsy (the kinds not specified) by the use of sugar, to the exclusion of all other aliments and every kind of medicine. Being himself attacked with hydrothorax, ascites, and general leucophlegmasia, a formidable combination, truly, he made trial of the saccharine treatment, which, after a perseverance of six months, procured a complete recovery. He took, during this time, several pounds of Muscovado sugar daily. M. Andral relates the history of a man fifty years of age, who, after having suffered from obstinate intermittent fever and enlarged spleen, together with progressing dropsy, had recourse to a diet of sugar, of which he took an enormous quantity, and at the same time associated with it a little broth. At the end of three months the dropsy had disappeared and his health was restored. The same author cites the cure of a young woman affected with a chronic disease of the stomach, for which she took a pound of sugar daily, and a cup of cold beef broth; this treatment was continued for a length of time.

M. Chossat, after studying the effects of an exclusively saccharine regimen, found that sometimes it favored the formation of fat, sometimes that of bile; and that its use was attended with constipation in the first case and diarrhœa in the latter. Milk, however, produces analogous effects on the intestinal canal.

The different kinds of alimentary substances are next examined under the several heads of lenitive or assuasive, analeptic, tonic, stimulant, temperant, specific, and special, as acting on a particular organ or apparatus. Under the head of lenitive aliments come garden vegetables, roots, and the muco-saccharine fruits. To these must be added feculent articles—mucilage united to sugar, starch, vegetable jelly, gluten, and even the oil which is in a state of minute subdivision in seeds. This class of aliments is adapted to the first stage of chronic disease following nearly subdued acute inflammation; also to active hemorrhage; to gout brought on by a too succulent food, and by stimulants; to chronic inflammation of the lungs, pleura, stomach, liver and kidneys, the exanthemata, and to chronic dysentery.

Analeptic aliments are those which, while repairing the waste of the organism, are at the same time lenitive. Foremost of this class is milk, which combines in itself the nutritive elements of butter or animal oil, casein, albumen, lactin or the sugar of milk, various salts, and water. Milk has many resemblances to chyle and blood, in its composition and properties, which could not fail to make it interesting *a priori* in a therapeutical point of view. In its alimentary effects it must vary according to the animal from which it is drawn, the habits of this animal, its mode of nourishment, and the proportions of constituent parts of the milk. We regret not being able to follow the author in his observations on the dietetic value and applications of milk, but in the limited space left for us we shall hardly be able to do more than specify the subjects successively treated of in the volume. Milk ranks in the first line, in gastralgia with atony. Ass's milk calms, as we are assured by Barras, the erethism in this case better than any other substance, either alimentary or pharmacological. In diarrhœa and dysentery goat's milk has been greatly praised, in those cases particularly in which there is an absence of gastric distress. Cold milk diluted with water is specially adapted to infantile diarrhœa, when the abdomen becomes suddenly tympanitic. The prompt and entire removal of bowel disease, and wasting away of the flesh in infants prematurely weaned, or when the supply of the mother fails, by putting them to the breast of a healthy nurse, must be known to every practitioner of any experience. The milk regimen is also very serviceable in the first stages of phthisis, constitutional syphilis accompanied with much emaciation, asthenic gout, albuminuria, dropsy, and chronic diseases of the skin accompanied with much irritation. With milk are associated fecula, bread, eggs, etc. Whey is both a drink and a lenitive aliment. It contains sugar, casein, lactic acid, and an animal matter which has been compared to osmazome. Its use is praised by the author in wounds, after surgical operations, acute and chronic ophthalmia, irritation of the generative organs, and the acute forms of syphilis.

Tonic alimentation includes all those meats which contain gelatinous, albuminous, and fibrinous principles in a state of elaboration superior to that in which they exist in fishes, and in animals, which form a part of the lenitive diet. These meats nourish without stimulating, by giving tone, and gradually increasing the strength of the organs.

Stimulating diet consists of a great number of aliments of the preceding group, to which exciting properties are added by the mode of preparation, by seasoning, and by the use of stimulating drinks. Red meats from adult animals, and game, are the basis of this alimentation, which is called for in defective appetite, an asthenic condition of the economy, threatened gangrene, the latter stage of epidemic continued and exanthematous fevers, and in certain forms of scrofula and scurvy. In temporary depression of the vital powers alcoholic liquors have a reviving and reanimating influence. Coffee takes high rank in the list of drinks, food, and condiments which form the stimulating diet. It carries off asthenic, or, as they are often called, nervous headaches, caused by cold and moisture, or by heat and moisture. It restores the sensibility which opium or excessive mental labor have greatly disturbed and obtunded. It is also serviceable in asthma, and in the asphyxia caused by charcoal, and in the nervous stage, or that of invasion of intermittent fever. It is administered on these occasions in a strong decoction, to which is sometimes added lemon-juice. Tardy menstruation in young persons of great susceptibility is also relieved by coffee.

This article has been used under circumstances in which no *a priori* reasoning would have suggested the idea, viz., in strangulated hernia. The author details a case of this nature. Taxis, ice, the internal use of belladonna had been used in vain. It was then that Dr. Durand, an old physician, eighty years of age, having accidentally heard of the case, told of his having seen similar ones treated in Havana, and of having himself treated many others with great success by the following plan: Give every quarter of an hour a cup of strong coffee, warm, and slightly sweetened; the infusion is to be made of about eight ounces of roasted coffee in twelve cups (size not stated) of boiling water. The last four cups are to be taken every half hour, in place of a quarter of an hour. This prescription was closely followed, and at the end of the fifth cup there was gurgling, and after the ninth cup the intestine was returned.

Temperant aliments contain but little nutritive matter, and are very watery; they are consequently distinguished from the other classes in their being directly cooling and antiphlogistic. The principles found in this group are vegetable acids united with jelly, mucilage, gum, fecula, and saccharine matter. A regimen of this nature will diminish the activity of the circulation, and increase the above discharges and the renal and other secretions; and by abating that of the skin it is opposed to plethora. These substances, in the form of drinks, are valuable adjuvants in diseases marked by plethora and phlogistic or bilious irritation—in typhus and yellow fevers, and hemorrhages.

Aliments endowed with specific properties form the next subject of notice. First of these come antiscorbutics, such as water-cress, nasturtium, veronica, radish, parsley, turnips, horse-radish, leeks, onions, garlic, chervil, ginger, celery, sorrel, and acid fruits, and especially lemons, and also raw potatoes; and among the drinks, beer, cider, Seltzer water, etc. Into the composition of most of these articles there enter an acrid essential oil and vegetable acids, united to mucilaginous and feculent principles. Experience shows that by these substances alone scurvy has been often cured. Many of them are used at the same time, and often with meat and wine. Taraxacum has proved to be a good antiscorbutic. Sugar has been given largely with no little success. Alimentary regimen alone, in due method and persevered in, will often suffice for the cure of scorbutic ulcers.

Much interest must be attached to the question, how far a particular organ or apparatus can be directly acted on or specially modified by certain aliments? The laxative operation of some of these substances is a familiar fact; and this knowledge ought to be turned to better account than it has been, in the treatment of constipation, a disorder which proves so often an annoyance and a detriment to many persons. On the other hand, we may avail ourselves of the astringent properties of other articles of food. Are there alimentary substances which act in a special manner on the pulmonary system? is a question asked by the author. The answer is given affirmatively in a qualified sense, but without that minuteness of specification which is so desirable. We may, it seems to us, be very sure that the prevention and cure of phthisis will be found eventually to turn on a suitably-devised alimentary regimen, more even than on the pneumatic trials on which more confidence has hitherto been placed. When we speak of pneumatic trials, we mean not only the inhalation of various gases and vapors, but all the atmospheric influences at work in the changes of season and of climate.

In reference to alimentary matters acting on the brain and nervous system, the author speaks of lettuce, both in its crude state and when cooked, as calming the sensibility and inviting to sleep. More evidently certain is a special action of a different kind produced by coffee on the brain, which it excites in an agreeable manner, with the production of wakefulness and a genial flow of thought. As such, it is adapted to prostrated states of the nervous system and the narcotism produced by opium. We do not dwell now on all the various circumstances under which it is contraindicated, such as a febrile state, nervous excitement, irregular and immoderate action of the heart, inflammatory dyspepsia, cutaneous diseases, etc.

We have not room for more than stating the subject of chapter fifth, in which is made the application of the principles that regulate diet in chronic diseases, specifying, in the first instance, chronic phlegmasiæ, the dermatoses, syphilis, and scrofula. In chronic inflammations of the alimentary canal, M. Herpin, who saw so much of these diseases in Algeria, is quoted as saying that pharmacological agents, though often useful, necessary, and even indispensable, occupy but the second rank. In the selection of alimentary substances, even in the same case, we shall be influenced by the season; lenitives and demulcents being preferred in the spring, and a tonic course in the autumn. In syphilis much of our success will turn on the hygienic treatment, which must be regarded as decidedly active. The author, on this subject as well as on scrofula and the scrofulous diathesis, enters into some minuteness of inquiry. His advice as to the means of checking obesity and removing leanness will interest many of his readers. Would that some of us of the Cassius kind could be changed in this latter particular!

The alimentary regimen in nervous affections is next discussed in an excellent spirit. So also is the following section on the circumstances which relate chiefly to external agents, and to the habits of the parties to whom we offer advice.

The second section, on the regulation of the respiratory functions for therapeutical purposes, opens with a chapter in which the operation of the properties of the atmosphere is investigated, under the respective heads of heat and dryness, heat and moisture, atmospherical vicissitudes, light, and electricity, and is concluded by remarks on the chemical properties of the atmosphere, regarded in a therapeutical point of view. On all these points we meet with much instructive commentary and apposite advice.

Changes of climate and changes of locality are the subjects of the two following chapters. The study of medical geography is enjoined in this connection; it is one in which every practitioner has a direct interest. Daily, at any time, as remarked by M. Boudin, the most retired country physician may be consulted and his opinion asked respecting the best place for a consumptive or a scrofulous patient to take up his abode. Ignorant of medical geography, the oracle thus consulted runs the risk of giving not only a doubtful response like that of Delphi of old, but a positively erroneous one, and sends the patient in a direction the very reverse of that which he ought to take. The prevention, and, in a good measure, the treatment of endemic diseases cannot be satisfactorily indicated without a knowledge of the effects of localities on the health of the inhabitants. A familiar example of this truth is seen in obstinate intermittent fevers, the only means of cure of which consist in travel and a change of locality. But of all diseases, phthisis pulmonalis is the one in which the most numerous trials have been made and the greatest hopes indulged, on the score of change of climate and locality. The author has not gone as fully into this subject as would be justifiable from the details accumulated during the last thirty years, nor has he adverted to the change of opinion which is now taking place respecting the hitherto universally accredited superiority of warm climates for the consumptive. Looking to the fact of perfect assimilation and hematosis being the main functional conditions for the prevention as well as cure of phthisis in its early stages, we cannot deny the superiority of cool and even cold climates for enabling nutrition and its attendant phenomena to be carried on. Just now medical opinion is in a state of transition on this subject. To show how far we are from having collected all the data from which to deduce guiding principles in the therapeutical application of climatic influences, we would mention the fact, that in Iceland, the climate of which is cold, raw, and changeable, and every way ungenial, consumption is a rare disease. This is the more surprising when we add that the compensating benefits of abundant and varied alimentation are wanting to the people of this island.

Travel forms the subject of a chapter which is filled with pertinent remarks. Sea voyages have not engaged the attention of the author. We quote one passage.

"Grief, and the concealed annoyances of married and family life, are, more frequently than the world supposes, the chief cause of deeply-seated disorders, at first of a nervous character, or without any appreciable lesion of moment, and afterward becoming more and more organic. The physician will be deceived in the nature of lesions of the heart, for example, if he do not go back to this source. Pharmacological and surgical means are useless, and either aggravate the evil or produce only a transient and deceptive effect. But when traveling is sometimes proposed, the suggestion is eagerly received and the invalid makes the avowal that his condition is owing to a void in the heart and to ennui. Traveling may become, at this time, a curative means, unless the powers of life are too much exhausted, or the advice comes too late."

Suggestive remarks and enforcing cases are found in a chapter on the secretions and excretions therapeutically considered; but the field of observation is now for the most part changed, and in place of physiological we have pathological phenomena and an exhibition of the relief of the latter by means more therapeutical or pharmacological than hygienic. In not a few cases it is true that suddenly suppressed eruption, giving rise to serious disease, may be restored by hygienic means, and all the functions resume their customary rhythm. We need not introduce examples in proof of this position: they must be familiar to every physician. If it were desired to make a study of any one secretion, the suspension and interruption of which are followed by the greatest number and variety of disorders, it would be the menstrual.

Instructive matter, albeit not new, is contained in a chapter on bed and clothing. The extreme importance of keeping or early taking to one's bed on the first premonition of many diseases, especially those of an epidemic character, cannot be urged too earnestly. In cholera, the result of the attack will mainly depend on an early precaution of this kind. In addition to the entire repose of the body in a recumbent position in bed, a uniform temperature of the skin is easily preserved and mild perspiration induced. Similar care is required at the termination of rheumatism, pulmonary inflammation, and in the exanthemata, and during incipient convalescence after these diseases. The sudden impression of cold on the skin has often been followed by relapse or alarming fatal sequelæ in patients convalescing from scarlet fever and measles. Frictions, as a powerful but too much neglected agent of therapeutical hygiene, find a chapter after the one just noticed.

In proportion to the advances we make in the work of M. Ribes are we met by increasing richness and variety of matter, and hence our regret that we are unable, for want of space, to do more than tantalize our readers by giving them the invoice, as it were, in place of a view of the things themselves. The third book treats of hydrotherapia in general; its first section is on ordinary hydrotherapia, or the common uses of water internally and externally; and also on special hydrotherapia, that which we commonly understand by hydropathy. The second section includes the subject of mineral hydrotherapia, or the use of mineral springs, in its hygienic relations. More than 260 pages are occupied in the discussion of these various topics—all of them of exceeding interest to the physician who would look beyond the contents of the apothecary's shop for an array of means furnished by nature, we ought to say a beneficent Providence, for the preservation of health and the prevention of disease. We are glad to have it in our power to offer some compensation to our readers for the present privation by pointing out to them the volumes of Dr. John Bell on Baths in their hygienic and medicinal applications, and on the Mineral Springs of the United States and Canadas. Contributing to the same end are the instructive works of Drs. Moorman and Burke on the Virginia Springs.

A new and large field for the exercise of intellectual and moral philosophy by the physician, in their application to the treatment of disease, through timely appeals to the affections, sentiments, and understanding of the invalid, is opened out in books third and fifth, with the latter of which the volume terminates. Beyond some few empirical trials, this field is scarcely cultivated at all by our medical men, who confound physiology of the brain with cloudy metaphysics; and who, because they have heard that the latter is unsatisfactory, and yields but little, infer that the former is equally unproductive, if not sterile. In whatever way opinion shall ultimately settle down respecting the merits of phrenology, as taught by Gall, its originator, and his able followers and commentators, Spurzheim and George Combe, it must, we think, be conceded that its study, more than that of any other body of mental philosophy, teaches a habit of intellectual analysis, and of noting the results, as they offer themselves in daily life, of the action of the innate faculties of the mind modified by the external circumstances of education, government, religion, and climate. We have advanced half way in the study of etiology if we remain ignorant of the power of the passions or the inordinate action of the propensities and sentiments, in giving rise to disease. So, also, we deprive ourselves of half the resources of therapeutics if we do not succeed in enlisting the aid of those passions, in their mitigated exhibition, for the cure of disease. Under both aspects daily opportunities are now offered. Would that they could have been entirely withheld by the absence of the fearful struggle which now convulses our once peaceful and happy country! A large record will be made by the number of intelligent and observing medical men in both of the antagonist sections of the United States.

The subject of the fourth book of the volume is one which has begun, of late years, to occupy general attention more than before. We refer now to physical exercises, under the head of gymnastics. The author examines them in their therapeutical application, and lays down rules for their employment. Too much importance cannot be attached to gymnastic exercises under the direction of an experienced and reflecting gymnast, as one great, perhaps the chief, means of arresting the physical deterioration to which the inhabitants of large cities are, of necessity, doomed, if no such correctives are introduced. In the enumeration of the different kinds of exercise, we do not find swimming, which, as it meets the double indications of bathing and of exercise, merits special consideration. As we are on this subject, we must not omit to express our gratification at the facilities, on a large scale, for all the gymnastic exercises and varieties of bathing, for both sexes, in the Physical Institute and Natatorium, under the direction of Mr. Hlasko, and his aid, Dr. Jansen.

In our regretfully abbreviated notice of M. Ribes's treatise on Therapeutical Hygiene we have restricted ourselves almost entirely to the task of analysis. However imperfect this has been, our readers cannot fail to see the wide range, and the variety of subjects discussed, and to acknowledge the great utility of their application, not only to the cure of disease, but also to their prevention. The reviewer, on the present occasion, would in earlier life have eagerly engaged in making a translation of this volume; and he does not abandon the hope, that in more propitious times than the present, some of his younger brethren will do this good service to the profession and to the general public.

